# Supplementary material for: Task-evoked deactivations: dissociation between BOLD fMRI and FDG
Source: bioRxiv. 2026 May 18:2026.05.14.725188. Preprint. [Version 1] doi: 10.64898/2026.05.14.725188 (PMC13228381; doi:10.64898/2026.05.14.725188)
Supplement: Supplement 1 [file NIHPP2026.05.14.725188v1-supplement-1.pdf]

bioRxiv preprint doi: https://doi.org/10.64898/2026.05.14.725188; this version posted May 18, 2026. The copyright holder for this preprint (which was not certified by peer review) is the author/funder, who has granted bioRxiv a license to display the preprint in perpetuity. It is made available under a CC-BY 4.0 International license.

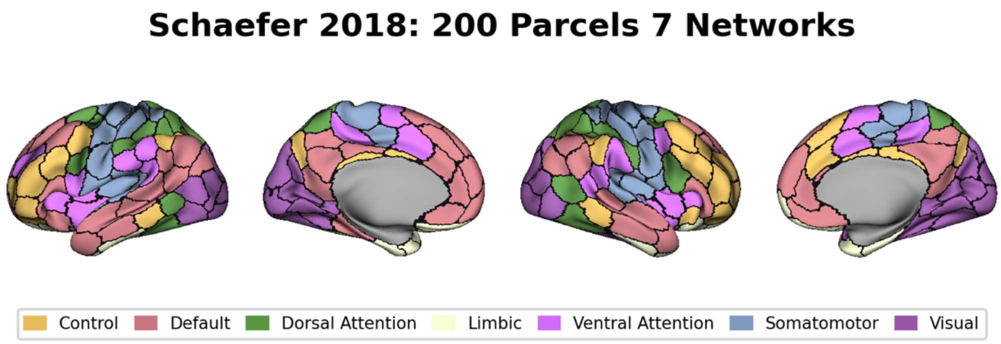

Figure S1: Network assignment of each of the 200 Schaefer parcels.

bioRxiv preprint doi: https://doi.org/10.64898/2026.05.14.725188; this version posted May 18, 2026. The copyright holder for this preprint (which was not certified by peer review) is the author/funder, who has granted bioRxiv a license to display the preprint in perpetuity. It is made available under a CC-BY 4.0 International license.

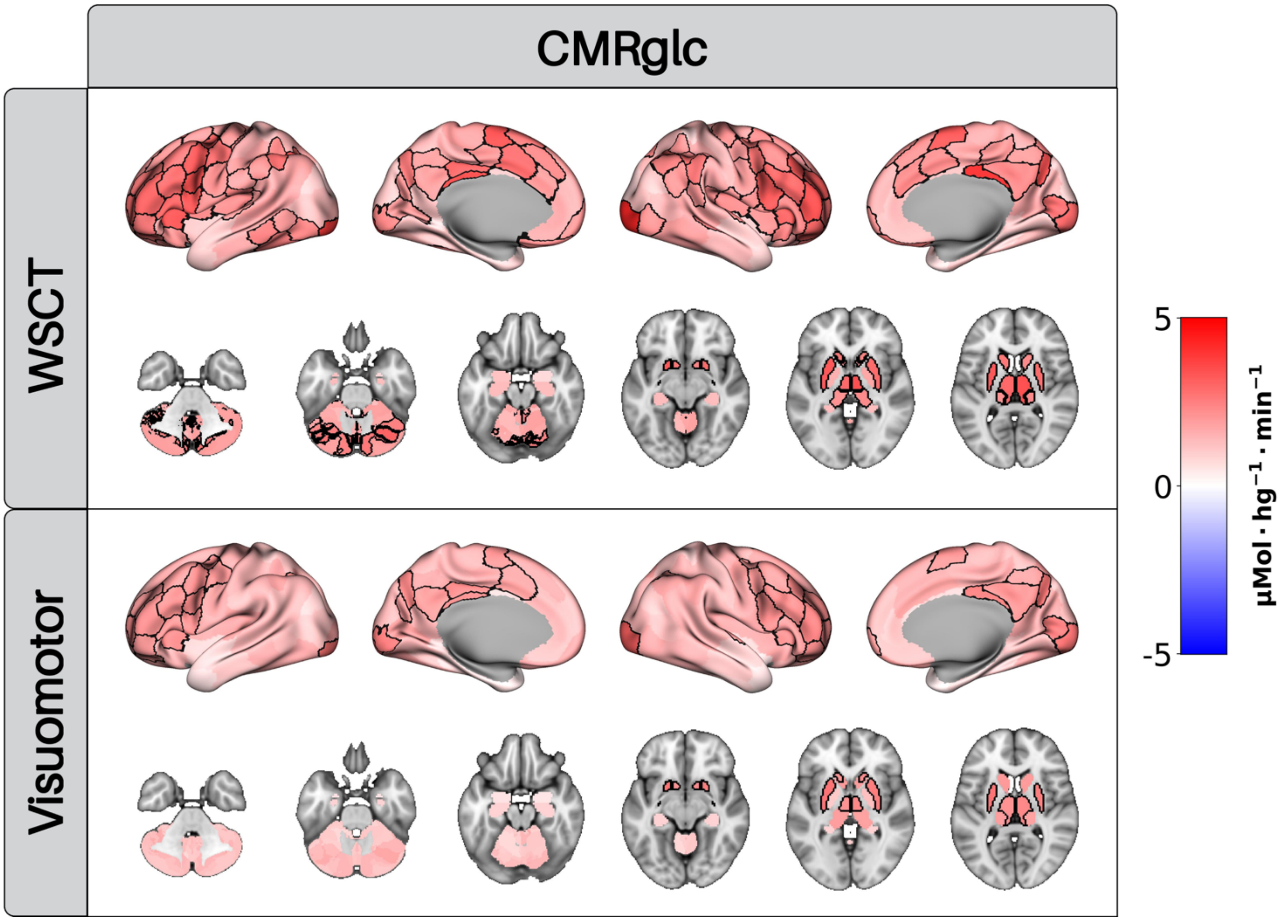

Figure S2. Lack of task-evoked decreases in CMRglc is robust to input function method. Same analysis as in **Figure 2** using an alternative method to extract the image-derived arterial input function (Sari et al., 2017). The results largely mirror those in **Figure 2**, with widespread increases in CMRglc observed in both tasks. There are differences between methods however: more regions met the 95%, particularly for the visuomotor task with this method, and the effect sizes differed, with larger effects for the visuomotor task and smaller effects for the WSCT.

bioRxiv preprint doi: https://doi.org/10.64898/2026.05.14.725188; this version posted May 18, 2026. The copyright holder for this preprint (which was not certified by peer review) is the author/funder, who has granted bioRxiv a license to display the preprint in perpetuity. It is made available under a CC-BY 4.0 International license.

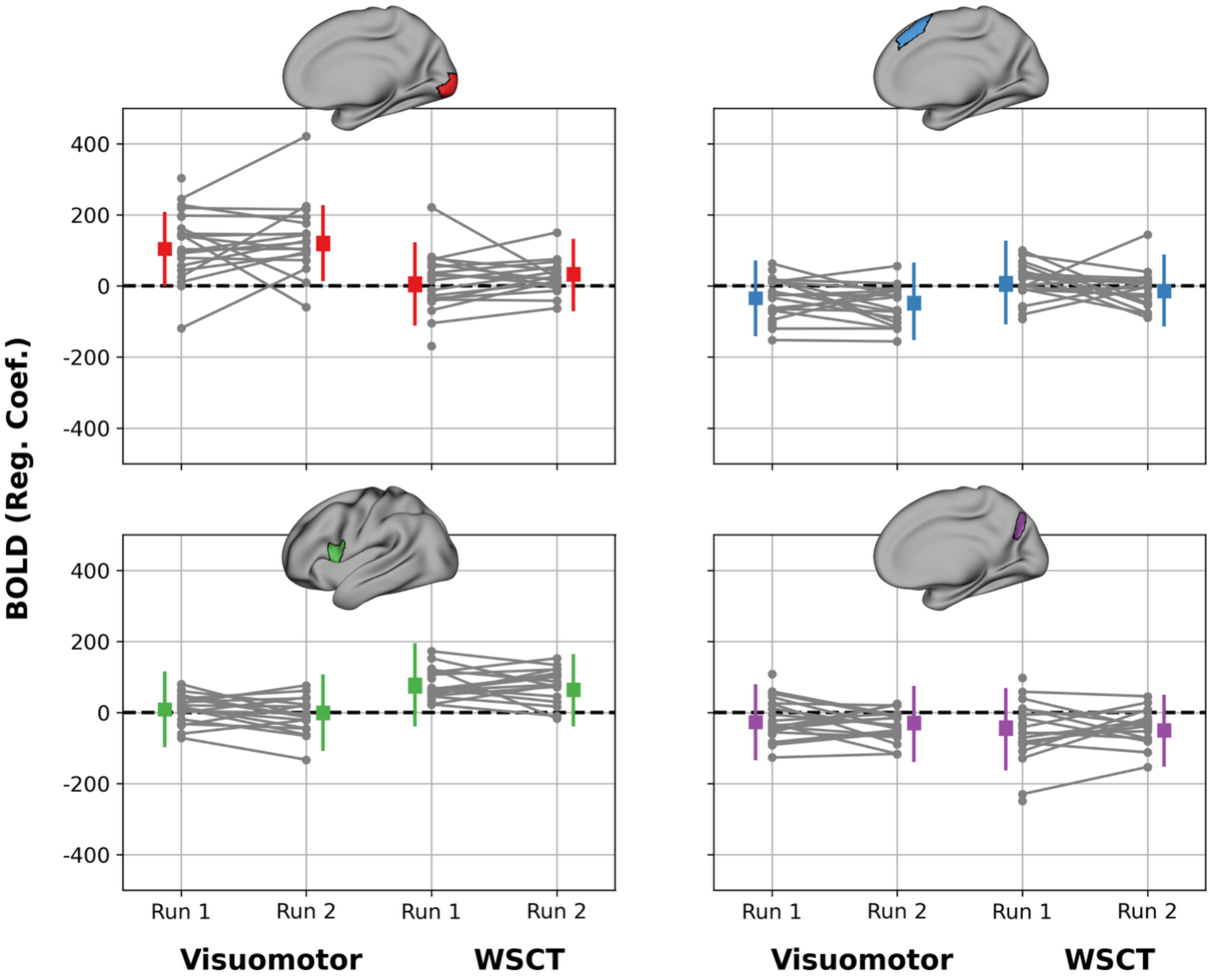

Figure S3: Estimate of task-evoked BOLD activity in four regions of interest. Each gray dot is from single BOLD run, with gray lines connecting data from individual participants. The thick colored lines are the 95% highest density interval (HDI) for each run, while the thin colored lines are the 95% posterior predictive interval. The only region where the HDI excluded zero between runs was the right medial visual cortex (top left), where the second run had a larger BOLD response than the first run during the WSCT.
